# Supplementary material for: Novel Microdialysis Technique Reveals a Dramatic Shift in Metabolite Secretion during the Early Stages of the Interaction between the Ectomycorrhizal Fungus Pisolithus microcarpus and Its Host Eucalyptus grandis
Source: Microorganisms. 2021 Aug 26;9(9):1817. doi: 10.3390/microorganisms9091817 (PMC8465077; doi:10.3390/microorganisms9091817)
Supplement: Supplementary file 1 [file microorganisms-09-01817-s001.zip › Figure S1.pdf]

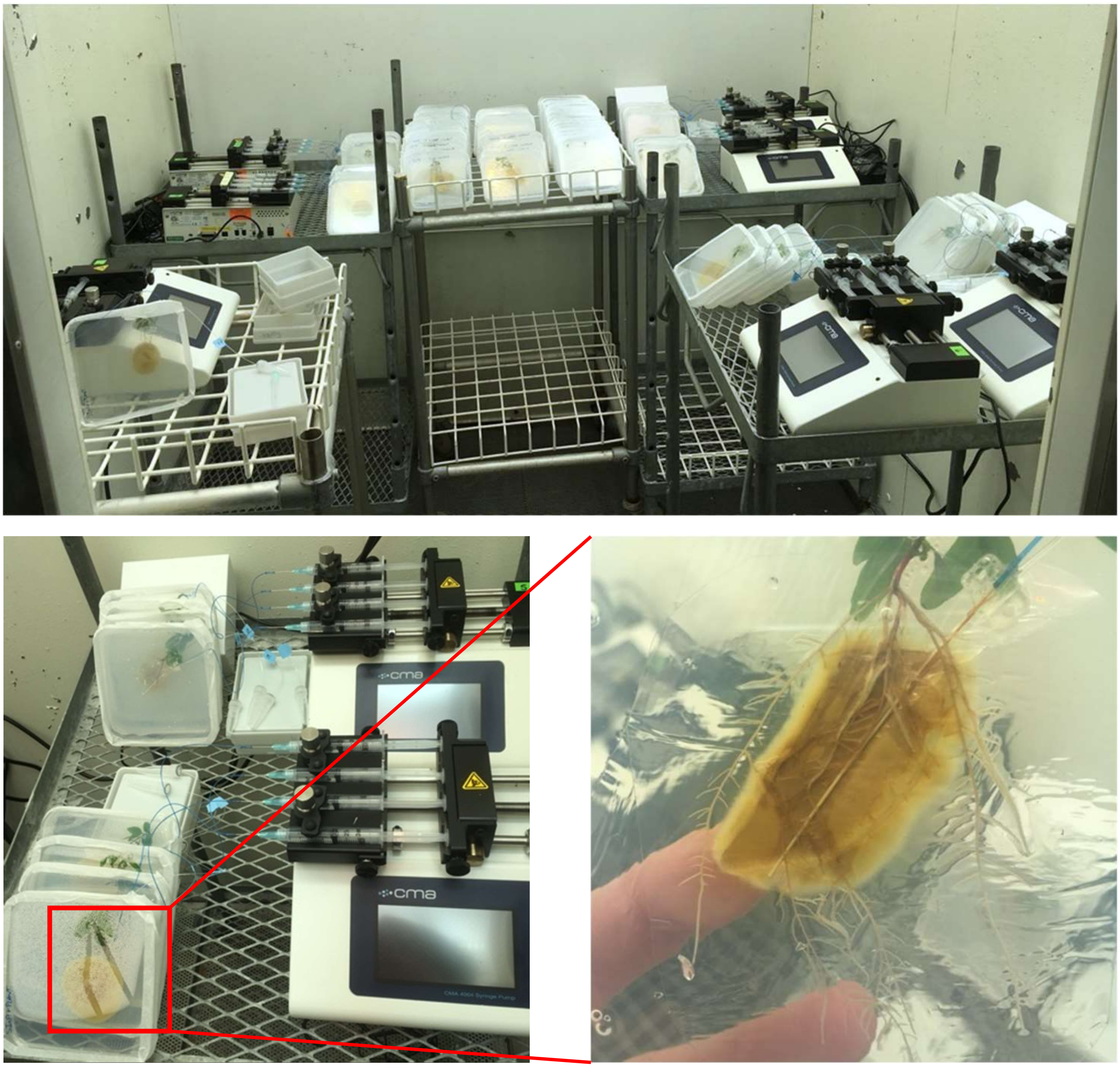

Figure S1: Experimental setup for sampling of metabolites of *E. grandis* and *P. microcarpus* using microdialysis probes.
