# Supplementary material for: Novel Microdialysis Technique Reveals a Dramatic Shift in Metabolite Secretion during the Early Stages of the Interaction between the Ectomycorrhizal Fungus Pisolithus microcarpus and Its Host Eucalyptus grandis
Source: Microorganisms. 2021 Aug 26;9(9):1817. doi: 10.3390/microorganisms9091817 (PMC8465077; doi:10.3390/microorganisms9091817)
Supplement: Supplementary file 1 [file microorganisms-09-01817-s001.zip › Tables S1-3.pdf]

Table S1. Results of principal components analyses (PCA) and orthogonal partial least squares discriminatory analyses (OPLS-DA) for 560 features measured with negative ion mode (ESI-).

Component	R2x(cum)	Eigenvalue	R2y(cum)	Q2(cum)
PCA				
1	0.677	17.6		0.638
2	0.771	2.45		0.727
OPLS-DA				
1	0.675	17.6	0.421	0.0292
2	0.769	2.42	1	0.538
Orthog. Comp. 1	na.			

Table S2. Results of principal components analyses (PCA) and orthogonal partial least squares discriminatory analyses (OPLS-DA) for 1530 features measured with positive ion mode (ESI+).

Component	R2x(cum)	Eigenvalue	R2y(cum)	Q2(cum)
PCA				
1	0.655	16.4		0.516
2	0.744	2.24		0.467
OPLS-DA				
1	0.564	14.1	0.413	0.262
2	0.604	0.986	1	0.662
Orthog. Comp. 1	0.141	3.53		

Table S3. List of top 20 putative molecular features associated with each condition (Fungus, Plant, Indirect Contact), sorted by variable-in-project scores from OPLS-DA analyses.

**Negative Mode**				
**RT (Mins)**	**m/z**	**VIP**	**Putative Class**	**Putative ID**
**Fungus**				
2.90	763.69	0.931	Unknown	Unknown
4.51	782.67	0.927	Unknown	Unknown
1.25	741.17	0.925	Unknown	Unknown
0.86	708.62	0.875	Unknown	Unknown
2.93	519.71	0.866	Unknown	Unknown
6.66	675.58	0.832	Unknown	Unknown
3.18	503.74	0.821	Unknown	Unknown
0.38	427.81	0.811	Unknown	Unknown
3.36	680.74	0.808	Unknown	Unknown
2.64	714.69	0.795	Unknown	Unknown
2.77	730.64	0.777	Unknown	Unknown
4.50	794.66	0.766	Unknown	Unknown
3.39	760.65	0.741	Unknown	Unknown
3.51	496.77	0.681	Unknown	Unknown
2.86	593.72	0.664	Unknown	Unknown
2.92	796.60	0.663	Phospholipid	Phosphatidylethanolamine alkyl 18:0-20:0
4.96	590.82	0.606	Unknown	Unknown
2.87	603.73	0.599	Unknown	Unknown
2.49	810.62	0.585	Lipid	Ceramide phosphate d19:0/26:0
2.75	605.69	0.522	Unknown	Unknown
**Plant**				
Na.				
**Indirect Contact**				
3.31	317.53	1.736	Unknown	Unknown
3.31	318.04	1.732	Unknown	Unknown
2.96	153.02	1.729	Unknown	Unknown
2.96	153.36	1.724	Unknown	Unknown
3.59	367.21	1.717	Lipid	Unknown
3.27	261.01	1.716	Unknown	Unknown
3.31	115.03	1.716	Unknown	Unknown
3.59	412.05	1.714	Unknown	Unknown
2.09	177.08	1.712	Quartenary Ammonium	Carnetinamide
3.12	433.02	1.709	Phenylpropanoid	Unknown
2.96	242.92	1.709	Unknown	Unknown
3.65	257.08	1.709	Unknown	Unknown
3.47	687.09	1.703	Nucleoside	Unknown

RT (Mins)	m/z	VIP	Putative Class	Putative ID
3.59	412.22	1.702	Unknown	Unknown
3.48	203.06	1.698	Unknown	Unknown
3.52	396.05	1.698	Unknown	Unknown
2.96	151.01	1.698	Unknown	Unknown
3.59	411.60	1.698	Phenylpropanoid	Flavonoid
3.31	274.05	1.697	Unknown	Unknown
3.31	317.18	1.694	Fatty Acid	*N*-dodecanoyl-hydroxyglycinate

Positive Mode

**Fungus**				
4.50	404.34	1.226	Fatty Acid	Unknown
3.62	641.04	1.208	Unknown	Unknown
3.50	1075.10	1.190	Unknown	Unknown
0.41	654.86	1.187	Unknown	Unknown
3.49	1106.06	1.186	Unknown	Unknown
0.41	758.82	1.186	Unknown	Unknown
4.29	493.08	1.177	Unknown	Unknown
0.43	379.84	1.177	Unknown	Unknown
1.18	152.16	1.158	Unknown	Unknown
0.43	630.77	1.152	Unknown	Unknown
3.46	489.03	1.151	Nucleotide	Guanosine-5'-phosphate-2',3'-cyclic phosphate
0.62	587.16	1.148	Azole	1-[4-(4-chlorophenyl)thiazol-2-yl]-2-(pentan-3-ylidene)hydrazine
0.99	304.09	1.146	Dipeptide	Unknown
4.10	671.07	1.146	Unknown	Unknown
4.00	685.08	1.145	Flavonoid	Unknown
2.71	709.25	1.142	Carboxamide	N(2)-[(2Z)-2-(2-amino-1,3-thiazol-4-yl)-2-(methoxyimino)acetyl]-N-butyl-L-serinamide
3.47	367.02	1.140	Unknown	Unknown
2.26	697.21	1.136	Diarylether	Ampicillin
0.68	170.44	1.136	Unknown	Unknown
3.80	450.06	1.135	Unknown	Unknown

**Plant**				
1.34	379.87	1.760	Unknown	Unknown
0.50	294.01	1.739	Organic Acid	2,2-dichloro-1-phenylcyclopropanecarboxylic acid
3.41	439.77	1.673	Unknown	Unknown
3.58	395.56	1.604	Unknown	Unknown
3.00	849.54	1.454	Unknown	Unknown
3.31	261.50	1.439	Unknown	Unknown
0.45	687.73	1.438	Unknown	Unknown

RT (Mins)	m/z	VIP	Putative Class	Putative ID
0.44	636.74	1.378	Unknown	Unknown
4.58	576.37	1.299	Carbohydrate	2-O-palmitoyl-3-O-[(2E,4S,6S,8S,10S)-2,4,6,8,10-pentamethylhexacos-2-enoyl]-2'-O-sulfo-alpha,alpha-trehalose
8.61	1024.67	1.294	Unknown	Unknown
0.44	484.79	1.230	Unknown	Unknown
8.74	567.36	1.219	Organic Acid	Unknown
3.31	499.00	1.217	Nucleotide	Unknown
8.62	475.28	1.206	Lipid	Lipid A
0.50	494.01	1.205	Nucleoside	Ethyl cytidine triphosphate
0.45	348.82	1.092	Unknown	Unknown
4.45	561.36	0.764	Glycerophospholipid	Unknown
3.13	647.37	0.728	Glycerophospholipid	Unknown
6.80	628.31	0.561	Unknown	Unknown
3.36	652.39	0.558	Unknown	Unknown
**Indirect Contact**				
3.63	565.15	2.226	Flavonoid	Unknown
2.79	1039.06	2.214	Unknown	Unknown
0.50	561.24	2.118	Alkaloid	Hydroxyisodolichantoside
0.50	543.24	2.077	Fatty Acid	Unknown
5.78	367.37	1.982	Lipid	Campestanol
3.95	454.12	1.925	Unknown	Unknown
3.09	432.56	1.897	Unknown	Unknown
3.09	432.86	1.879	Unknown	Unknown
3.49	513.04	1.850	Organic Acid	Unknown
4.12	471.12	1.796	Unknown	Unknown
2.79	629.15	1.790	Carbohydrate	Unknown
6.75	607.39	1.721	Unknown	Unknown
3.51	460.04	1.689	Unknown	Unknown
2.45	274.04	1.680	Unknown	Unknown
0.50	517.22	1.643	Unknown	Unknown
3.16	496.06	1.642	Unknown	Unknown
5.35	583.36	1.582	Unknown	Unknown
3.60	412.04	1.568	Phenylpropanoid	{2,6-dimethoxy-4-[3-oxo-3-(2,4,6-trihydroxyphenyl)prop-1-en-1-yl]phenyl}oxidanesulfonic acid
3.04	110.04	1.553	Unknown	Unknown
6.94	608.39	1.538	Prenyl Lipid	Ganoderic acid
